# Feasibility of smart wristbands for continuous monitoring during pregnancy and one month after birth

**DOI:** 10.1186/s12884-019-2187-9

**Published:** 2019-01-17

**Authors:** Kirsi Grym, Hannakaisa Niela-Vilén, Eeva Ekholm, Lotta Hamari, Iman Azimi, Amir Rahmani, Pasi Liljeberg, Eliisa Löyttyniemi, Anna Axelin

**Affiliations:** 1 Department of Nursing Science, University of Turku, FI-20014 University of Turku, Turku, Finland; 2 Department of Obstetrics and Gynecology, University of Turku and Turku University Hospital, FI-20014 University of Turku, Turku, Finland; 3 Faculty of Communication Sciences, University of Tampere, Tampere, Finland; 4 Department of Future Technology, University of Turku, FI-20014 University of Turku, Turku, Finland; 5 Department of Computer Science, University of California, Irvine, USA; 6 Department of Computer Science, Institute of Computer Technology TU Wien, Vienna, Austria; 7 Department of Biostatistics, University of Turku, FI-20014 University of Turku, Turku, Finland

**Keywords:** Activity tracker, Biosensor, Feasibility, Internet-of-things, Pregnancy, Self-monitoring, Smart wristband, User experience, Wearable sensors

## Abstract

**Background:**

Smart wristbands enable the continuous monitoring of health parameters, for example, in maternity care. Understanding the feasibility and acceptability of these devices in an authentic context is essential. The aim of this study was to evaluate the feasibility of using a smart wristband to collect continuous activity, sleep and heart rate data from the beginning of the second trimester until one month postpartum.

**Methods:**

The feasibility of a smart wristband was tested prospectively through pregnancy in nulliparous women (*n* = 20). The outcomes measured were the wear time of the device and the participants’ experiences with the smart wristband. The data were collected from the wristbands, phone interviews, questionnaires, and electronic patient records. The quantitative data were analyzed with hierarchical linear mixed models for repeated measures, and qualitative data were analyzed using content analysis.

**Results:**

Participants (*n* = 20) were recruited at a median of 12.9 weeks of gestation. They used the smart wristbands for an average of 182 days during the seven-month study period. The daily use of the devices was similar during the second (17.9 h, 95% CI 15.2 to 20.7) and third trimesters (16.7 h, 95% CI 13.8 to 19.5) but decreased during the postpartum period (14.4 h, 95% CI 11.4 to 17.4, *p* = 0.0079). Participants who could not wear smart wristbands at work used the device 300 min less per day than did those with no use limitations. Eight of the participants did not wear the devices or wore them only occasionally after giving birth. Nineteen participants reported that the smart wristband did not have any permanent effects on their behavior. Problems with charging and synchronizing the devices, perceiving the devices as uncomfortable, or viewing the data as unreliable, and the fear of scratching their babies with the devices were the main reasons for not using the smart wristbands.

**Conclusions:**

A smart wristband is a feasible tool for continuous monitoring during pregnancy. However, the daily use decreased after birth. The results of this study may support the planning of future studies and help with overcoming barriers related to the use of smart wristbands on pregnant women.

**Electronic supplementary material:**

The online version of this article (10.1186/s12884-019-2187-9) contains supplementary material, which is available to authorized users.

## Background

The monitoring of pregnancies is needed to secure the health and wellbeing of a pregnant woman and her unborn baby. Currently, health care staff perform this monitoring during regular appointments in maternity care units [1]. With the support of modern technology, such as the Internet of Things (IoT), continuous monitoring, tracking, and transmitting personal health metrics in real time has become possible in more advanced ways than ever before [2–4]. This evolution has also given rise to opportunities for maternity care [5–7], as IoT connects devices (e.g. smart wristbands) remotely to servers, thus enabling the monitoring and data analytics through Web-based user interfaces from anywhere and at any time [8]. It is well known that IoT solutions have not reached their full potential by adapting to health care [2].

Monitoring technology has been shown to promote health variables (e.g., physical activity) in pregnant women [9, 10]. Ideally, self-controlled monitoring would engage pregnant women in their health care better than ever before. This could save resources in maternity care, for example, by decreasing the number of visits or by detecting possible problems. Identifying methods of promoting and measuring health in high-risk mothers, for example in those with an increased risk of gestational diabetes, is another possible application of this technology. Collected health data may help health care staff to follow up with their patients and to make personalized care decisions. The tracked data may also be used to automatically recognize high-risk patients for additional checkups or interventions [3, 4]. Therefore, health care staff and the research community need feasible, valid, and reliable measurement tools [11].

Wearable technology, such as smart wristbands, has tremendous potential in maternity care; however, it also poses challenges [3]. One of the biggest concerns is sustaining patients’ long-term engagement with this modern technology [12]. To overcome this challenge, it is essential to understand the needs and barriers of the target population, so that device feasibility and acceptability can be determined in an authentic context [3, 13]. Currently, IoT devices are usually assessed with usability testing and case studies, but feasibility analysis in health care is rarely conducted [2]. The implementation of wearable technology, such as smart wristbands, in maternity care, requires an understanding of mothers’ perspectives of and adherence to these methods.

The aim of this study was to evaluate the feasibility of smart wristbands in collecting continuous activity, sleep and heart rate data from nulliparous pregnant women from 13 weeks of gestation (gwk) until 1 month postpartum. We examined the actual use of the devices (wear time), use-associated factors, satisfaction with the devices, expressed interest, and self-perceived behavioral changes.

## Methods

### Study design

The feasibility of smart wristbands was tested in a prospective observational feasibility study with nulliparous pregnant women [14]. The recruitment took place between May and September 2016, and the data collection ended in June 2017.

### Participants and recruitment

Twenty pregnant nulliparous women were recruited during their first trimester ultrasound examinations at two maternity clinics in Southwest Finland. The eligibility criteria for participants were (a) ≥18 years of age, (b) ≤15 gwks, and (c) a singleton pregnancy. Women who did not understand Finnish or who did not have smartphones or computers compatible with the smart wristband were excluded. Twenty participants were estimated to permit the assessment of feasibility in daily-life activities in the target population, as well as the estimation of a sample size for further trials involving using smart wristbands in pregnancy [14].

The health care staff at the maternity clinics provided initial written and verbal information about the study. All eligible women (*N* = 22) were interested in participating in the study and spoke with the research staff by telephone. During each telephone call, the researcher provided more detailed information about the study and a face-to-face meeting was appointed if the woman was willing to participate. Two women declined to participate after receiving the study information over the phone due to work-related restrictions against wearing a smart wristband. Twenty women met the researcher and provided written informed consent. Smart wristbands and instructions for using them were given to the participants at the meeting. The women were asked to wear the wristbands continuously from the recruitment to 1 month postpartum, as removing the device increases the likelihood of forgetting to use it.

### Smart wristband as a measurement tool

Several factors influence the selection of a smart wristband for a long-term maternal monitoring study [15, 16]. First, the weight, size, and degree of comfort of a wristband play key roles in increasing the wear time. Moreover, an appropriate human-device interaction, such as an onboard display and an interactive mobile application, could encourage the participants to wear the device [17]. Sufficient battery capacity and internal data storage are also important in enabling longer intervals between device charging and data synchronization [18].

Garmin Vívosmart HR (Garmin Ltd, Schaffhausen, Switzerland) smart wristband was chosen from the available, affordable price, devices due to its small size, smooth design on straps, waterproofness, and ability to estimate both steps and heart rate. It integrates a biosensor and an activity tracker that is available for consumers. This small (21 mm x 12.3 mm) and light (29.6 g) smart wristband estimates steps, distance (based on steps), used calories, heart rate, stairs climbed, intensity of physical activity, and total hours of sleep, sleep levels, and sleep movement. The data are collected continuously and are synchronized to the Garmin Connect website or the Garmin Connect app. The rechargeable battery can last up to five days on a single charge. The charging of the battery takes two hours. Various factors (e.g., screen brightness and vibration alerts) may shorten the battery life in between charges [19]. In the healthy non-pregnant population, Garmin Vivosmart has demonstrated good validity in measuring step counts when worn during slow walking speed [20] and a heart rate in rest, but it underestimates the heart rate when the intensity of exercise progresses [21].

### Data collection and outcomes

**Fig. 1:**
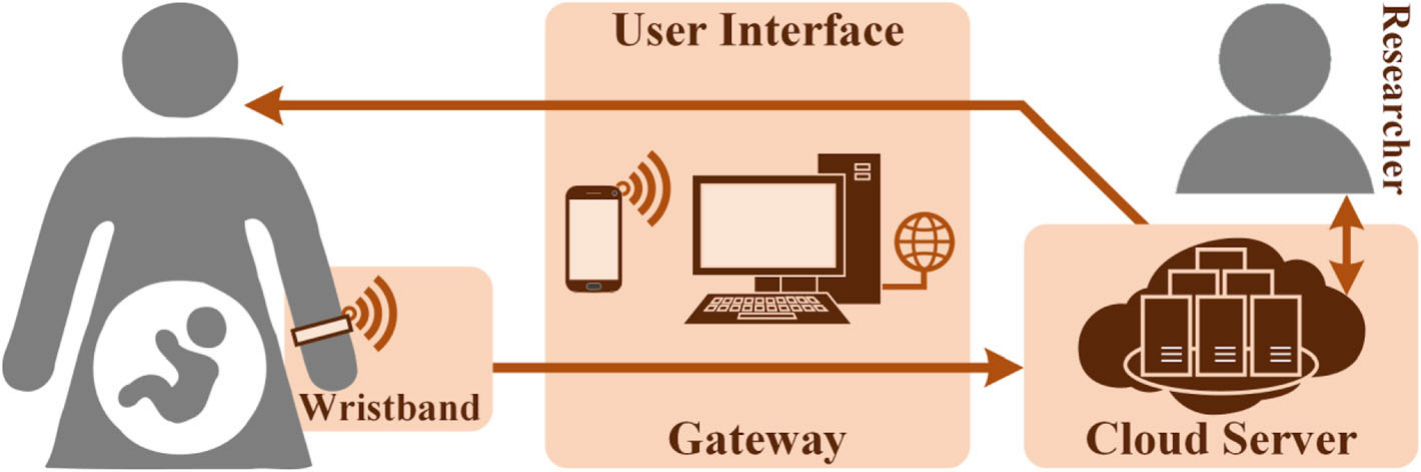
IoT-based maternal monitoring system

The main outcome was the actual use (wear time) of the smart wristband during a seven-month follow-up period during pregnancy and postpartum. The smart wristbands were included in an IoT-based system where several sensing, communication, and computing resources were exploited. The data collected with the wristband were transmitted to the servers through a gateway device, which was a smartphone or a computer (Fig. 1). The participants were asked to synchronize the data once a day or while charging the smart wristbands. The data were accessible to researchers and participants throughout the study via interface devices (e.g., smartphone). The wear time of the device was considered to be data available in the server.

When recruited, all participants completed a questionnaire for background information. The participants’ experiences with the smart wristband (satisfaction, expressed interest, and self-perceived behavioral changes) were investigated with phone interviews that the research staff conducted. Each participant was interviewed nine times during the study; twice during the first month of the data collection; and once a month thereafter. If a participant was not reached by phone, a text message was sent to ask for a convenient time for a follow-up call. The phone interviews included multiple-choice and open-ended questions [see Additional file 1]. In addition, pregnancy and birth-related data were collected from the participants’ electronic patient records.

### Data analysis

To describe and summarize the participants’ background information and the wear time of the devices, adjusted means, confidence intervals, median, and range were used as continuous variables, and counts with proportions were used for categorical variables. Inter-rater agreement between the objective wear time and the self-reported wear time was examined by calculating Cohen’s kappa coefficient. For this, the objective wear time was categorized for each trimester and postpartum period (All the time: 7 days/week ≥ 20 h/day; Several days/week: ≥ 3 days/week; Once a week: ≥ 1 day/week; Not at all: 0 h/week). The data collection covered 24 hours per day. Furthermore, the amount of valid wake time data was analyzed with the criterion of 10 hours of step count data at wake time per day for at least four days a week [22, 23].

To understand in greater depth the actual use of the smart wristband, we investigated whether the pregnancy weeks were associated with the wear time of the device. Therefore, we used a hierarchical linear mixed model with repeated measures including one within-factor measure (time as categorical). Compound symmetry covariance structure was used for time. Analyses were performed both weekly and based on trimesters of pregnancy. All tests were performed as two-tailed test with the significance level set at 0.05. The analyses were performed using SAS System, Version 9.4, for Windows (SAS Institute Inc., Cary, NC, US).

The responses to multiple-choice questions in the interviews were analyzed using descriptive statistics and the open-ended questions with qualitative content analysis [24].

### Research ethics

The Ethics Committee of the Hospital District of Southwest Finland and the University of Turku (35/1801/2016) approved the study protocol. The smart wristbands were purchased for the study, and the permission to use Garmin Vivosmart HRs was obtained from the manufacturer, Garmin Ltd. In addition, written informed consent was obtained from all participants. Following the completion of the study, the smart wristbands were handed over to the participants as an incentive.

## Results

### Participants

**Fig. 2:**
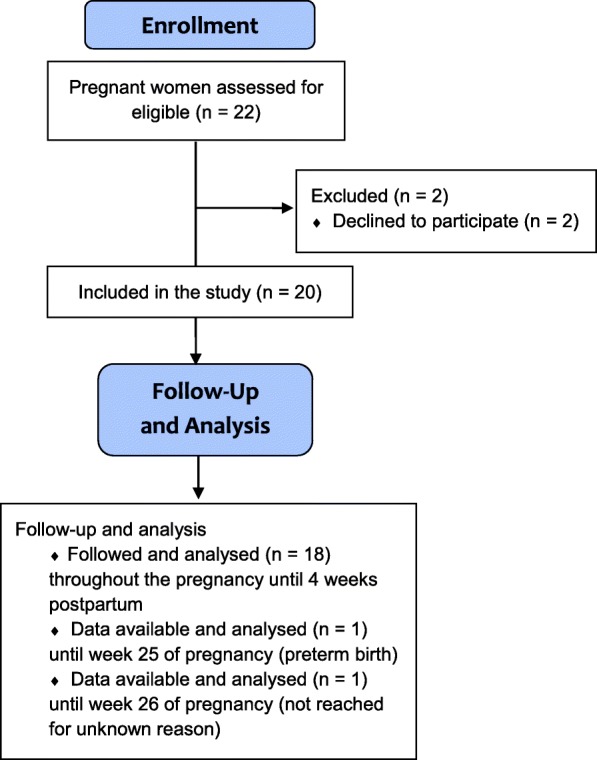
Flow chart of enrollment

Twenty pregnant women participated in the study. The flow of the participant enrollment is described in Fig. 2. Participants’ median age at recruitment was 24 years (ranging from 18 to 37 years). The median pre-pregnancy body mass index (BMI) of the participants was 24.4 kg/m^2^ (a range of 17.7–43.5 kg/m^2^). For 18 of the participants, the follow-up lasted until one month postpartum. One participant delivered prematurely and could not be reached after the onset of pregnancy complications at 25 gwks, and another participant could not be reached for an unknown reason after 26 gwks. The participant-related characteristics are described in Table 1.

**Table 1:** Descriptive statistics for participant-related variables

Characteristics of participants (*n* = 20)	Median (range)/Mean (SD)/n (%)
Age, years	
Median (range)	24 (18–37)
Pre-pregnancy BMI^a^, kg/m^2^	
Median (range)	24.4 (17.7–43.5)
Weeks of gestation at recruitment	
Median (range)	12.9 (7.6–15.0)
Marital status, n (%)	
• Married or living with a partner	17 (85%)
• Single	3 (15%)
Highest educational qualification, n (%)	
• Below secondary education	4 (20%)
• Secondary education	9 (45%)
• College or polytechnic	4 (20%)
• University	3 (15%)
Employment status, n (%)	
• At work	10 (50%)
• Unemployed	2 (10%)
• Student	5 (25%)
• Entrepreneur	3 (15%)
Step counts per day during pregnancy	
Mean (SD)	5576 (1808)
Gestational diabetes, n (%)	5 (25%)
Smoking during pregnancy, n (%)	5 (25%)
Weeks of gestation at delivery	
Median (range)	40.4 (29.1–41.7)

^a^ *BMI* body mass index

### The actual use of the smart wristband

The participants used the devices for 3259 days out of 4270 potential days during the study period, which comprised 76% of potential days. This resulted in a median use of 182 days (range 18–222) per participant during the seven-month study period. The duration of the pregnancy had an impact on the study period.

**Fig. 3:**
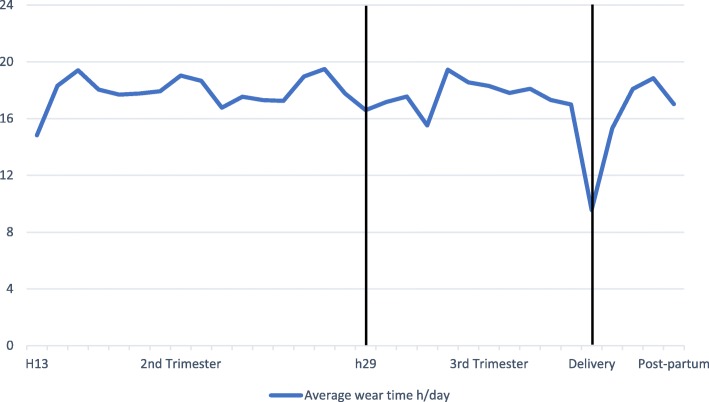
Average wear time (h/day) of the smart wristband during the seven-month follow-up

The wear time of the devices did not change from the second to third trimesters (*p* = 0.28) rather, it decreased at postpartum (*p* = 0.0079), being 17.9 hr, (95% CI 15.2 to 20.7), 16.7 hr, (95% CI 13.8 to 19.5), and 14.4 hr (95% CI 11.4 to 17.4) per day, respectively. The decrease on wear time from second to third trimester was 1.3hr (95% CI -1.1 to 3.6), third trimester to postpartum period 2.3hr (95% CI -0.3 to 4.8), being overall 3.5hr (95% CI 1.0 to 6.1) from second trimester to postpartum. The average daily wear time in each gwks is described in Fig. 3. The devices were used more when the participants were awake; 66% of the wear time was awake time. The detailed wear times during the follow-up period are reported in Table 2.

**Table 2:** Measured and self-reported wear time of the smart wristband during pregnancy and after delivery

Weeks of pregnancy/postpartum	Second trimester, weeks 13–28 *n* = 20	Third trimester, weeks 29–41 *n* = 18	Postpartum, weeks 1–4 after delivery *n* = 13
Wear time, h/day			
Adjusted mean (95% CI)	17.9 (15.2–20.7)	16.7 (13.8–19.5)	14.4 (11.4–17.4)
• Min (h/day)	4.3	4.9	1.7
• Max (h/day)	23.6	23.7	23.8
Self-reported wear time			
• All the time	15 (75%)	14 (78%)	9 (69%)
• Several days/week	5 (25%)	4 (22%)	4 (31%)
• Once a week	0	0	0
• Not at all	0	0	0
The agreement between objective and self-reported			
Wear time^a^	Slight	Fair	Moderate
κ (*p-value)*	0.13 (0.412)	0.38 (0.05)	0.514 (<0.001)

^a^ For the inter-rater agreement the objective wear time was categorized for each trimester and postpartum period as follows: All the time: 7 days/week ≥20 h/day; Several days/week: ≥ 3 days/week; Once a week: ≥ 1 day/week; Not at all: 0 h/week

When only the valid awake data were included, 2777 days of data were available (65% of 4270 potential days). During the second trimester, the data were received for 1520 days (35.6% of the data, n = 19), during the third trimester for 1023 days (23.9% of the data, n = 17), and 232 days (5.4% of the data, n = 12) postpartum.

The number of participants actively wearing the smart wristbands decreased during the follow-up according to the data obtained from the smart wristbands. Three participants in the third trimester and eight participants at postpartum did not wear the device or wore it only occasionally; thus, 13 out of 20 participants continued to use the smart wristbands throughout the study period.

Based on the telephone interviews, most of the participants (*n* = 15, 75%) reported wearing the devices constantly during the second trimester, 14 participants (70%) in the third trimester, and 9 (69%) at postpartum. None of the interviewed women reported not using the device at all. The agreement with the objective and the self-reported wear time increased during the follow-up period.

### The factors associated with the use of smart wristbands

Five participants were not able to use the smart wristbands during their working hours. These participants used them approximately 300 minutes less per day compared with those with no use limitations. Furthermore, the week of gestation was associated with wear time (<0.001). The wear time in 13 gwks was lower than that during the rest of the gwks (16/27 of the weekly comparisons, *p* < 0.05). Furthermore, the wear time in the delivery week was significantly lower than in other weeks (all *p*- values < 0.001). For example, difference between 13 and 29 gwks was 1.8 hr (95% CI -1.1 to 4.7), between 29 gwks and the delivery week was 7.0 hr (95% CI 4.0 to 10.0), and finally between 13 gwks and the delivery week was 5.3 hr (95% CI 2.2 to 8.4).

More than half of the women (n = 11, 55%) had issues with charging the device (e.g., the device was forgotten in the charger, the battery had run out, or the charger was missing). Eight of the participants (40%) reported at some point of the long study period that the smart wristbands were uncomfortable to wear, especially at night. The wristbands irritated the skin, possibly due to pregnancy-related swelling. Based on four responses, forgetting to use the device was found to be a limitation. Changes in normal routines, such as holidays, poses a challenge with remembering to wear the smart wristband. Two participants did not use the smart wristbands in the hospital while giving birth. Three of the participants found wearing the smart wristband to be inconvenient while handling their babies and were worried about whether the devices might scratch their babies.

### Satisfaction with the device

Overall, participants perceived the smart wristbands to be easy and comfortable to use. The adequacy of the instructions given at recruitment was perceived as good. The results of the questions on functionality, wearability, and the need for assistance are reported in Table 3.

**Table 3:** Participants’ experiences with the smart wristband

	First follow-up call, 2 weeks after the recruitment *n* = 20	Second trimester, weeks 13–28 *n* = 20	Third trimester, weeks 29–41 *n* = 18	Postpartum, weeks 1–4 after delivery *n* = 18
Total number of calls	20	57	55	20
Mean number of calls/participant	1	3	3	1.7
Functionality, mean (SD) *(1 = very difficult – 5 = very easy)*	4.5 (0.6)	4.6 (0.5)	4.6 (0.51)	4.2 (1.2)
Wearability, mean (SD) *(1 = very uncomfortable − 5 = very comfortable)*	4.0 (0.9)	4.1 (0.6)	4.2 (0.6)	3.9 (1.3)
Needed assistance with the smart wristband, n (%)				
No	16 (80%)	15 (75%)	18 (100%)	17 (94%)
Yes	4 (20%)	5 (25%)	0	1 (6%)
Parameters of interest, n (%)^a^				
• Steps	19 (95%)	20 (100%)	18 (100%)	10 (56%)
• Quality of sleep	15 (75%)	15 (75%)	11 (61%)	8 (44%)
• Heart rate	12 (60%)	15 (75%)	11 (61%)	5 (28%)
• Calorie consumption	3 (15%)	7 (35%)	6 (33%)	4 (22%)
• Something else	3 (15%)	8 (40%)	8 (44%)	4 (22%)
• Not following any	0	0	0	5 (28%)

^a^The participants were allowed to select multiple response options

Participants provided some negative feedback on the validity of the measures. Eight participants perceived the device underestimating the number of steps due to the immobility of the hand (e.g., when carrying things or pushing a baby in a pram). Sometimes the women detected that the intensity minutes or the stairs climbed were not registered correctly. Six participants mentioned the overestimation of time spent sleeping. One participant considered the heart rate measurement to be unreliable.

Difficulties in synchronizing the smart wristband with their phones, tablets, or computers were quite common (*n* = 11) during the long follow-up period, even though the device should synchronize itself in regular intervals via the Bluetooth. Other technical issues were related to software updates, sudden power shutdowns, and the change of a smartphone or computer. In most cases, the participants solved the problems by themselves, whereas some reported asking for help from their spouses (*n* = 5) or from a researcher (*n* = 2).

### Expressed interest and self-perceived behavioral changes

During the second trimester, all of the participants were interested in the step counts, and most of them (*n* = 15) were interested in their quality of sleep and heart rate. The expressed interest toward all parameters decreased as the study progressed; for example, only half of the participants were interested in their step counts after giving birth (Table 3).

According to the interviews, the impact of the smart wristbands on the participants’ behavior was conflicting. Almost all of the participants (*n* = 19) stated at some point during the long follow-up period that the smart wristbands did not have any impact on their behavior. The self-perceived impact on behavior was stronger at the beginning of the pregnancy compared with in the third trimester and at postpartum. However, 13 participants reported that the smart wristbands had motivated them to increase physical activity to reach their daily step goals. Four women observed the intensity of activity based on their heart rates from the smart wristbands. They reported checking to make sure that their heart rates did not rise too high. Some of the participants reported that they were able to detect coming down with the flu by observing their heart rates. A few participants (*n* = 2) used sleep data to get enough sleep.

Blood sugar levels, blood pressure, diet (caloric intake), and their babies’ heartbeats were reported as measures of interest when the participants were asked what other information they would have liked to know. However, the possibility of detecting a fetal heartbeat subsequently raised some questions about increased stress.

## Discussion

This study indicated that a smart wristband is a feasible tool for monitoring continuous data during pregnancy. However, challenges exist, such as being prohibited from wearing the device at work, and technical problems, which need to be taken into consideration. Even though pregnant women wore the smart wristbands well during pregnancy and experienced them in a mostly positive way, the number of participants wearing the smart wristbands and the wear time decreased as their pregnancies progressed. This decrease became even more evident after giving birth. The results of this study increase the understanding of possible reasons not to use the wristbands during pregnancy and after delivery.

Recruiting participants in the study was straightforward and the pregnant women were eager to use the smart wristband. Regardless of the decrease of the wear time as the follow-up progressed [25], almost all of the recruited women committed to the study. Notably, the self-reported wear time was higher than the objective wear time. The self-reported data were, however, collected retrospectively. Pregnancy is a window of opportunity for a woman to change her lifestyle to one that is healthier, for example, by focusing on physical activity [26], and therefore; pregnant women are a favorable target group for continuous monitoring. The women in this study used the smart wristbands more than the participants did in the only available longitudinal study of pregnant women, which reported an average of 100 days of the use of a wristband during pregnancy [27]. The discrepancy might be explained by the fact that in Huberty et al.’s (2016) study, the participants were blinded to the data. In the present study, the participants actively followed their data from the screens of the devices and the application. If the data would not have been available, this might have diminished the compliance and, in turn, the wear time.

Our results indicated that the gwk was associated with wear time. The first week of data collection (week 13 of pregnancy) with the smart wristbands lacked more data than the other weeks did. A person might spend the first week with smart technology trying to learn how to use the device and remember to wear it every day. Furthermore, the number of participants wearing the smart wristband decreased during the postpartum period. Recovery from the delivery and adaptation to a new phase in life focusing on the newborn possibly accounts for the decrease. In addition, changes in routines (e.g., holidays [25]) decreased the use of the smart wristbands. These are important to note when planning studies with short data collection periods, especially if trying to collect data from specific trimester of the pregnancy or postpartum period. To avoid data loss during data collection, the instructions for using a device, and the possibility of asking for assistance from the researchers should be carefully planned. It is notable that some workplaces, such as hospitals and restaurants, may limit the use of smart wristbands for hygiene reasons. Even though this is essential to consider when planning a study sample, the data collected from these women might have value even with the data loss that occurs during their work hours.

Technical problems, forgetting to use the device, and the perceived poor reliability of the data are important issues to consider in studies using smart wristbands. Regardless of the careful evaluation of the design of the device and the comfort of wearing it prior to data collection, some participants still reported feeling discomfort when wearing it. The reported discomfort will require attention in studies using smart wristbands, even though this might be partially avoided by including instructions on how to clean the device to avoid skin irritation to the research information [18]. During the postpartum period, some of the women were concerned with whether the device might scratch their babies. In future studies, this could be avoided by instructing women to place soft fabric wristbands over their devices while handling their babies.

These results were partly in accordance with previous findings because discomfort [17], physiological reactions (e.g., skin irritation [18]), or dislike of the design [17, 28] were reasons for decreasing the wearability of the devices. Inversely, the ease of use [17] and long battery life [17, 29] are known to be important to users. The perceived reliability issues with the activity trackers are also known to impact the use of a device [13, 28, 30]. This was also the case in this study, especially regarding the limited recording of step counts during activities when hand movements were restricted (e.g., carrying something or pushing a pram). This problem would have been solved by educating participants on the principles of accelerometer functions [31].

During pregnancy, the participants were mostly interested in following data linked with their health behavior, such as the step count data, sleep parameters, and heart rate. In our study, the mean daily step of 5576 was less than the reported 7000 steps per day in the non-pregnant Finnish female population (20 to 39 years old) [32]. Interestingly, two-thirds of the participants reported perceiving some positive effects on behavior during the seven-month study period. However, almost all of the participants reported that the effects were not permanent. The result is consistent with studies on non-pregnant participants [12, 25] and suggests that the motivational impact of activity trackers does not last for a long period of time and that a device itself cannot be deemed an intervention. In addition, the participants in this study expressed interest in other parameters outside of the used smart wristband, which should be considered in future studies. Monitoring fetal-related issues might work as a motivational factor for using the device.

When planning a study including technological devices, the researcher also needs to take into consideration the rapid progress of the technology. For example, during this study, the smart wristbands that are available to consumers have changed, as one of the manufacturers has exited the wearable technology market.

### Strengths and limitations

The strengths of our study were the long follow-up period and the combination of qualitative and quantitative approaches. Another strength of our study was the inclusion of risk pregnancies, as these groups may need additional monitoring and guidance during pregnancy. The small sample of only 20 participants limit the generalizability of the results of this study. However, for a feasibility study, a small sample is considered to be sufficient [14]. Furthermore, the nulliparity, and the singleton pregnancy can be seen as a limitation. Women with no prior children might have more time to concentrate on their pregnancies, as they do not yet have any children to look after.

Our study seems to be among the few studies investigating the feasibility of any kind of smart wristband during pregnancy with a long follow-up period. Only one longitudinal study that involved the use of a smart wristband throughout pregnancy and that focused on physical activity in pregnancy was found [27]. The follow-up time in other pregnancy-related studies was short, ranging from three to 14 days at different time points during pregnancy (e.g., [6, 7, 33]). The reporting acceptability of smart wristbands can guide the development of future study designs to take into account the reasons for adherence or non-adherence in field-based studies [34].

## Conclusion

The actual use of a smart wristband during pregnancy was found to be good. However, the wear time significantly decreased in the postpartum period. The continuous monitoring, tracking, and transmitting of personal health metrics in real time using wristbands in maternity care is a feasible possibility. The design and comfort of such a device need to be carefully evaluated. Our results may be utilized in future research and development projects that use wristbands as a measurement tool or as part of an intervention during a woman’s pregnancy.

## Additional file

Additional file 1: Questions used in the phone interviews. This file provides the multiple-choice and open-ended questions used in the phone interviews. (DOCX 13 kb)

## Abbreviations

BMI: Body mass index
IoT: Internet of things
