# Supplementary material for: Feasibility of smart wristbands for continuous monitoring during pregnancy and one month after birth
Source: BMC Pregnancy Childbirth. 2019 Jan 17;19:34. doi: 10.1186/s12884-019-2187-9 (PMC6337833; doi:10.1186/s12884-019-2187-9)
Supplement: Supplementary file 1 — Questions used in the phone interviews. This file provides the multiple-choice and open-ended questions used in the phone interviews. (DOCX 13 kb) [file 12884_2019_2187_MOESM1_ESM.docx]

| 1. How clear was the device manual?  (Response options from 1=not clear at all to 5=very clear) 2. How much did you use the device over past month? (Response options: all the time, several days in a week, once a week or less, none at all)   If you did not use the device, please, specify the reason.   1. How was the functionality of the device over the past month? (Response options from 1=very difficult to 5=very easy) 2. How was the wearability of the device over the past month? (Response options from 1=very uncomfortable to 5=very comfortable) 3. What were the possible problems with wearing or using the device over the past month?      1. Have you needed any assistance with your device over the past month and why? (Response options: yes/no) If yes, what problems did you have, and who assisted you with your device?      1. What parameters have you followed with the smart wristband over the past month? (Response options: steps, quality of sleep, heart rate, calorie consumption, something else, none) 2. What kind of impact has the smart wristband had on your daily life during the past month? 3. What parameters would you like to follow during your pregnancy, if you could choose whatever you wanted? |
| --- |
